# Supplementary material for: Conditional chemoconnectomics (cCCTomics) as a strategy for efficient and conditional targeting of chemical transmission
Source: eLife. 2024 Apr 30;12:RP91927. doi: 10.7554/eLife.91927 (PMC11060718; doi:10.7554/eLife.91927)
Supplement: Figure 3—source data 3. [file elife-91927-fig3-data3.zip › Figure 3 source data3 part 2.zip!/Figure 3-Source data 3-part2/1062-13-CRISPResso_Report_jt28fhr9.zip!/CRISPResso_on_1062_13/9.gcaggaaatgagctacctac.Alleles_frequency_table_around_sgRNA_gcaggaaatgagctacctac.pdf]

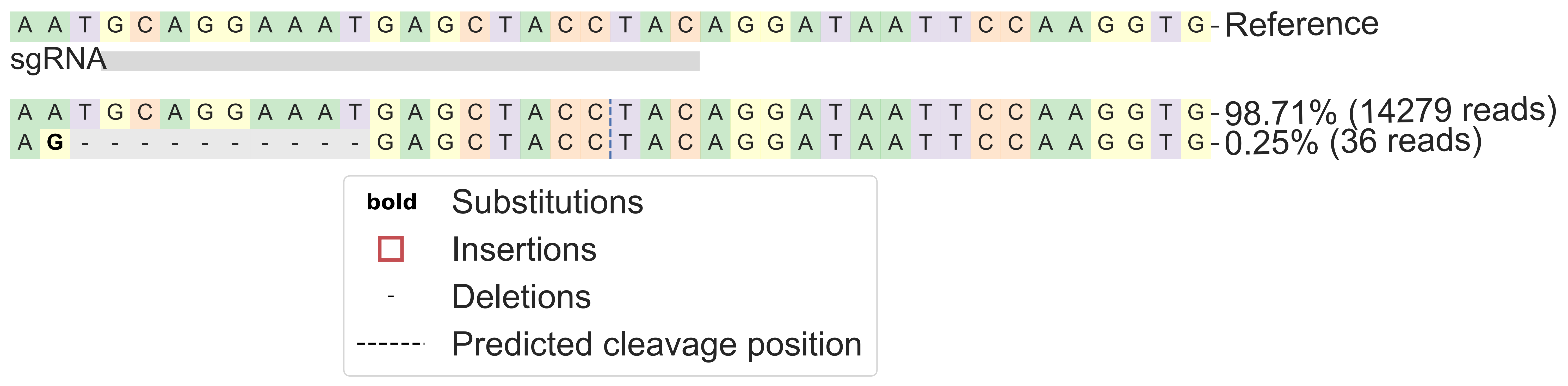
A A T G C A G G A A A T G A G C T A C C T A C A G G A T A A T T C C A A G G T G - Reference
sgRNA
A A T G C A G G A A A T G A G C T A C C T A C A G G A T A A T T C C A A G G T G - 98.71% (14279 reads)
A G - - - - - - - - - - G A G C T A C C T A C A G G A T A A T T C C A A G G T G - 0.25% (36 reads)
bold Substitutions
Insertions
- Deletions
Predicted cleavage position
